# Modification of Different Pulps by Homologous Overexpression Alkali-Tolerant Endoglucanase in *Bacillus subtilis* Y106

**DOI:** 10.1038/s41598-017-03215-9

**Published:** 2017-06-12

**Authors:** Meimei Wang, Jian Du, Daolei Zhang, Xuezhi Li, Jian Zhao

**Affiliations:** State Key Laboratory of Microbial Technology, Shandong University, Ji-nan City, 250100 Shandong Province China

## Abstract

Cellulase (mainly endoglucanase, EG) has been used in pulp modification for improving paper quality through environmentally friendly process. But low activity in alkaline pH and high filter paper activity (FPA) were still obstacles for extending the cellulase application in papermaking industry. In the study, an alkali-tolerant EG gene of *Bacillus subtilis* Y106 was homologous over-expressed for obtaining suitable enzyme used in pulp modification. The engineering strain could produce the crude enzyme with more alkali-tolerant EG and little PFA. Potential of the crude enzyme in modification of different pulps were investigated. It was found that the enzyme could be used for improving drainage and strength properties of pulps from softwood, hardwood and non-wood materials, especially non-wood pulp such as wheat straw pulp. The underlying mechanisms of pulp modification and different effects on various types of pulps by the EG treatment were also discussed by studying the change in fibers characteristics and fiber bonding.

## Introduction

Chinese paper industry uses a multiple fibrous raw materials including woods (hardwood, softwood), waste paper, and non-woods materials (wheat straw, rice straw, reed, bamboo, etc.), and nowadays the proportion of woods, waste paper and non-woods materials was about 22%, 63% and 15%, respectively^1^. The pulping methods used in the industry include chemical pulping, mechanical pulping, and chemi-mechanical pulping. Physical properties of paper were main priority in papermaking industry, which depend on fiber characteristics of raw materials and pulp types. There were lots of differences in physical properties of pulps from various raw materials and different pulping processes, which were often affected by pulp fibre properties (for example, fibre structure, chemical compositions, fiber length and width, etc.). For example, fibers in hardwood pulp were shorter and thicker than fibers in softwood pulp, thus hardwood pulp has relative lower strength properties, but higher bulkiness and absorbability. Problems existed in wheat straw pulp were low drainage, high fragility and low strength, which were probably related to high hemicellulose content, short fiber length and large number of fines in the wheat straw pulp^2^. Softwood pulp has high tensile index and folding endurance due to its long fibers, but the pulp beating process consumed more energy because the softwood pulp was hard to be refined compared to wheat straw pulp^3^. As for improving pulp properties, such as drainage of straw pulp, beating of softwood pulp, etc. different methods have been developed to adapt various types of pulps. Chemical process and mechanical process can fulfill the demand of pulp improvement, however, large chemicals and energy input will exert environment and economy more pressures^4^. On the other hand, paper production is an energy-intensive process^5^. Therefore, attempting to look for some new processes that could improve pulp properties and lower energy consumption is very important for improvement of paper quality and development of papermaking industry.

Enzymes-assisted modification comply such restrictions that lower costs and environmentally friendly for improving the strength properties of pulp. Some studies have reported that pre-treating pulp with cellulase resulted in a reduction in refining energy for same target drainage index^6, 7^. However, it should be noted that the cellulase treatment usually caused loss of fiber intrinsic strength, which affected tear strength of pulp in a negative way^8^. Cellulase was complicated and included Endo-1, 4-glucanases (EG, EC 3.2.1.4), cellobiohydrolases (CBH, EC3.2.1.91), β-glucosidase (EC3.2.1.21), etc. EG was an essential component for enzymatic degradation of cellulose. The EG particularly act on less ordered cellulose^9^, and CBH degrade preferentially crystalline cellulose in a progressive manner. Thus, EG made less damages to pulp properties and more suitable for pulp modification compared to the cellulase with high filter paperactivity (FPA) that contained CBH caused excessive degradation of cellulose^10–12^. On the other hand, paper production was usually carried out in neutral or alkaline environment, which urged more alkaline or alkali-tolerant enzymes to fulfill the demands. Therefore, cellulase component (mainly EG), which could affect the pulp properties^13^, and its pH-tolerance should be paid more attention for industrial application as well.

Many studies about pulp modification by EG treatment have been reported. For example, Hilden *et al*. (2005) used the endoglucanase Cel5A (pH 5.0) from *Trichoderma reesei* and endoglucanase (Novozym 476^TM^ fromNovozyme A/S, pH 5.0) from *Aspergillus*. sp to evaluate as probes for the surface properties of softwood- and hardwood chemical pulp fibres, and expounded the correlation between enzyme properties and fiber materials^14^. Mansfield *et al*. (1996) proved that the action of cellulase preparation on different fractions of Douglas fir Kraft pulp could decrease the defibrillation and reduce the fibre coarseness^15^. While Pere *et al*. (1995) found that endoglucanases have the ability to decrease the pulp viscosity with a lower degree of hydrolysis^16^. Oksanen *et al*. (2000) treated the recycled kraft pulps with cellulase (EGI, EGII) from *Trichoderma reesei* and found that EGs treatment significantly improved pulp drainage already at low dosage levels^17^. However, some problems such as low enzyme activity in alkaline range of pH, unreasonable enzymatic system such as high filter paper activity (FPA), limited the cellulase application in pulp modification. Producing the enzyme that more suitable for pulp modification should be further investigated by many researchers in the fields of papermaking, microbiology, enzymology, etc.

In our previous work, an alkali-tolerant strain *Bacillus subtilis* Y106 was screened from alkaline soil^18^. The crude enzyme from *Bacillus subtilis* Y106 was purified and the characteristics of the pure enzyme have been studied^19^. The Y106-*eg* gene (endoglucanase, GenBank: AF355629.1) from the genome of *Bacillus subtilis* Y106 has been cloned and overexpressed in *Escherichia coli* (*E*. *coli* JM109 Pc66)^20^. Our previous works focused on screening the alkali-tolerant enzyme and studying the crude enzyme’s modification effect on wheat straw pulp^18, 21^. In this study, we attempted to obtain the enzyme with high EG activity and low FPA in alkaline pH value by homologous expressing alkali-tolerant endoglucanase for promoting the commercial applicability of *Bacillus subtilis* Y106. Using three types of pulps from softwood, hardwood and non-wood respectively as substrates, we evaluated the potential of the recombinant EG in the pulps modification and discussed possible causes about the differences in enzymatic modification of different pulps.

## Results and Discussion

### Expression of recombinant EG and enzymatic characteristics

**Figure 1:**
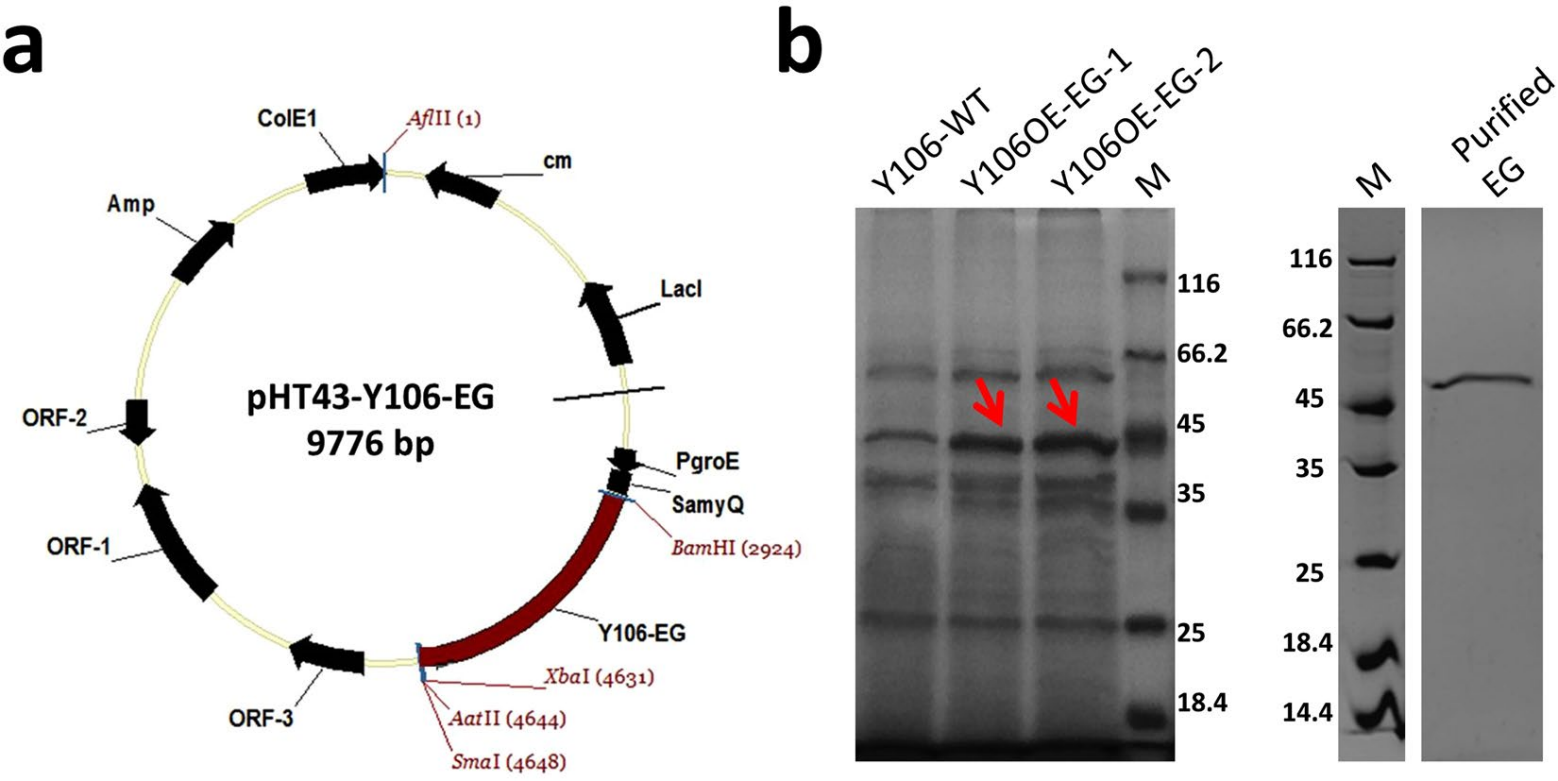
(**a**) Map of expression vector pHT43-Y106-EG (sub-cloning the alkali-tolerant Y106-*eg* in a shuttle vector pHT43), (**b**) Proteins detected by SDS-PAGE. Y106-WT: Crude enzyme of *Bacillus subtilis* Y106; Y106OE-EG-1/Y106OE-EG-2: Crude enzyme of *Bacillus subtilis* Y106OE-EG; Purified EG: EG purified from crude enzyme of *Bacillus subtilis* Y106OE-EG, M: protein molecular weight markers (kDa).

Using constructed recombinant vector pHT43-Y106-EG (Fig. 1a), the recombinant strain Y106OE-EG was screened out. The strain was cultured in the bran medium, and the fermentation broth was analysis by SDS-PAGE. Figure 1b showed that there was a protein band of 55 kDa in the electrophoretogram of engineering strain Y106OE-EG and wild type strain Y106-WT respectively, which were the same as the molecular weight of the purified recombinant EG. The protein intensities were analyzed using software imageJ 1.42q and obviously found that the bands of recombinant EGs from transformants Y106OE-EG-1 and Y106OE-EG-2 were particular darker than that of the EG from Y106-WT. The proteins were identified using mass spectrometry and proved that the alkali-tolerance EG was successfully and correctly over-expressed in *B. subtilis* Y106OE-EG.

**Figure 2:**
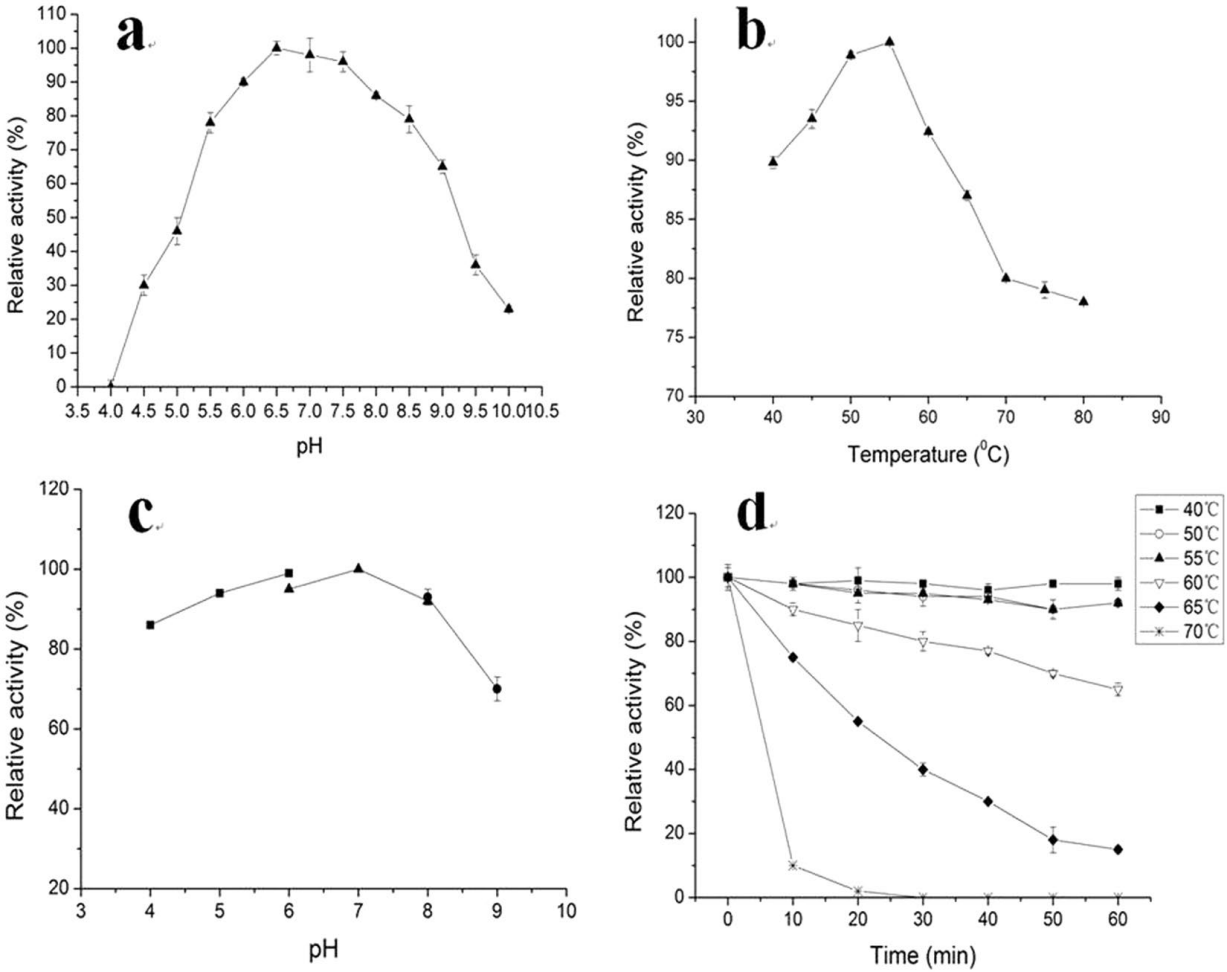
Effect of pH and temperature on the activity and stability of recombinant EG. (**a**) Effect of pH on activity, (**b**) Effect of temperature on activity, (**c**) pH stability, (**d**) Temperature stability. *The buffers used in the Figure 2a and c were as follows: sodium acetate buffer (pH4.0–6.0, ■), sodium phosphate buffer (pH 6.0–8.0, ▲), and Gly-NaOHbuffer (pH 9.0–10.0, ●).

The activity of purified recombinant EG from engineering strain Y106OE-EG was assayed against the pH and temperature. It was found that the optimum pH and temperature for the recombinant EG was pH 6.5 (Fig. 2a) and 55 °C (Fig. 2b) respectively. Figure 2c showed that the enzyme was stable at a broad range of pH (4.0~9.0) and 60–65% of the maximum activity could be kept at pH 9.0. The recombinant EG had a high activity at the range of 40–55 °C, and maintained more than 60% of the maximum activity at 60 °C for 1 h (Fig. 2d). It was indicated from the enzyme characteristics that the recombinant EG had good potential in alkali-environment of papermaking industry. It was also found that the characteristics of the recombinant EG were similar to that of the EG from wild strain Y106-WT^20^.

### Production of recombinant EG by engineering strain *Bacillus subtilis* Y106OE-EG in shake flask

**Figure 3:**
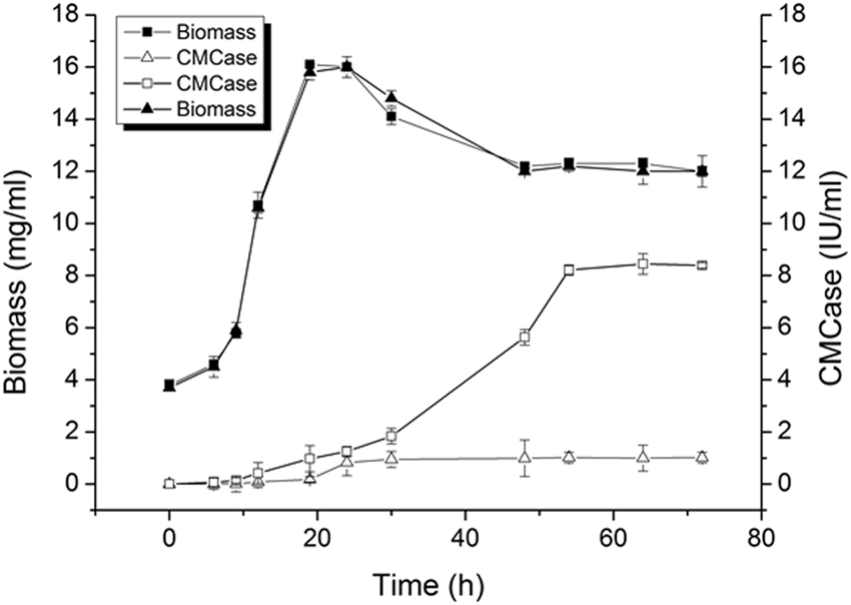
Changes of biomass and CMCase activity of engineering strain *Bacillus subtilis* Y106OE-EG and Y106-WT during liquor fermentation of 72 h in shaker flash culture (300 ml). ■ biomass of Y106OE-EG, □ CMCase activity of Y106OE-EG, ▲ biomass of Y106-WT, △ CMCase activity of Y106-WT.

Figure 3 showed the changes in biomass and CMCase (carboxymethylcellulase) activity between engineering strain Y106OE-EG and strain Y106-WT during the liquor fermentation time of 72 h. It was shown the similar trend in biomass between Y106OE-EG and Y106-WT. It increased obviously in the exponential growth phase (6 h to 18 h) when cultured in shaker flash, and stayed from 18 h to 24 h in lag phage of growth period, then decreased after 24 h, came with a cell death period.

CMCase activity increased with the biomass in exponential growth phase and lag phage, and still progressively increased after 24 h of fermentation time, though decreased in biomass, indicating that strain Y106OE-EG could continuously secret EG enzyme in entire fermentation process. The Y106-*eg* gene was controlled by the inducible promoter P*grace* ^22^ in engineering strain. After adding 0.1 mM IPTG into the culture media at 6 h of fermentation time (Biomass 0.6–1, OD600), CMCase activity of Y106OE-EG grew rapidly during 20 h to 50 h of fermentation time, which reached the maximum at 64 h of fermentation time. However, for strain Y106-WT, the CMCase activity of fermentation liquor just showed a slow growth from 20 h to 64 h. After 64 h of fermentation time, the CMCase activity of the crude enzyme produced by engineering stain Y106OE-EG was 8.45 ± 0.02 IU/ml, about 8.28 times higher than that of the original strain Y106-WT (1.02 ± 0.04 IU/ml). The FPA of the crude enzyme from engineering strain Y106OE-EG was also assayed and found it was extremely low (only 0.12 ± 0.004 IU/ml).

Studies showed that high FPA in crude enzyme was disadvantage for pulp modification because more degradation of cellulose especially crystalline cellulose was occurred by the synergy effect of EG, CBH and β-glucosidase (revealled by FPA activity), which led to fiber strength loss and pulp yield decrease^23–25^. On the other hand, most of commercial cellulase enzymes were from fungus and were vigorous in acidic environment. The characteristics of high FPA and low activity in alkaline environment became obstacles for their application in pulp industry. Some literatures reported that the EG with lower FPA from bacteria, particularly *Bacillus*. sp such as *Bacillus* sp. No. 1139, *Bacillus* sp. KSM-19, KAM-64 and KSM-520 and so on^26–29^, could be used in the wider range of pH value. But the EGs activity was too low to apply in industrial process. Some studies have also focused on promoting the alkaline or alkali-tolerant enzyme activity, for example, Deka *et al*. (2013 a, b) enhanced extracellular alkaline cellulase production from *Bacillus subtilis* by optimizing physical parameters and the CMCase activity was promoted from 0.43 IU/ml to 0.56 IU/ml at pH 6.0^30, 31^. Ariffin *et al*. (2008) optimized the liquor fermentation conditions of *Bacillus pumilus* EB3 and the CMCase activity of crude enzyme reached to 0.076 IU/ml at pH 7.0^32^. However, EG activities of the reported alkaline or alkali-tolerant cellulases were still low and could not meet industrial demand. The cellulase from engineering stain Y106OE-EG constructed in this paper had higher EG activity in a wide range of pH but low FPA, which was more suitable for pulp modification process. Besides, the EG could be rapidly produced during fermentation and the CMCase activity reached the maximum at 64 h of fermentation time. All the characteristics indicated that the cellulase from engineering stain Y106OE-EG has a good potential in pulp modification.

### Enzymatic modification of different pulps using the crude enzyme from engineering strain *Bacillus subtilis* Y106OE-EG

#### Effect of enzyme dosage

The bio-modification studies were performed using three different types of pulps such as aspen CMP (Chemi-mechanical pulp), pine CMP, and wheat straw CP(Chemical pulp), which stand for hardwood pulp, softwood pulp, and non-wood pulp respectively. Effects of enzyme dosages (0.0 IU/g, 0.20 IU/g, 0.60 IU/g and 0.80 IU/g) on physical properties of the pulps were shown by testing the beating degree, tensile index, burst index and tear index. As can be seen in Table 1, the values of beating degree (°SR) of all types of pulps decreased with the increased enzyme dosages, suggesting that treatment with the recombinant EG could improve the pulps drainages. The efficient enzymatic treatment could reduce beating degree of about 1.0 °SR for aspen CMP and 0.5 °SR for pine CMP at the same enzyme dosage of 0.60 IU/g compared to the control respectively. As for wheat straw CP, the recombinant EG treatment cut down about 3.0 °SR of beating degree at a lower enzyme dosage of 0.20 IU/g when compared with the control. Table 1 also showed that treatment with the recombinant EG of Y106OE-EG at 0.60 IU/g of enzyme dosage, promoted the tensile index from 15.48 N·m/g to 17.20 N·m/g for aspen CMP, meanwhile, the burst index (0.85 ± 0.04 kPa·m^2^/g) and tear index (1.77 ± 0.04 mN·m^2^/g) reached the maximum value at this enzyme dosage. There was negligible difference(*p* > 0.05) in pulp properties of aspen CMP between treated samples by the recombinant EG at a lower enzyme dosage of 0.20 IU/g and the control. However higher enzyme dosage (for example 0.80 IU/g) would not be in favor of the pulp strength properties except that the beating degree of enzymatic treated pulp was slightly decreased compared to the control. Strength properties of pine CMP treated by the recombinant EG at optimum enzyme dosage of 0.6 IU/g showed a similar variation trend, but improvement effect was slight compare to aspen CMP and wheat straw CP.

**Table 1:** Effect of enzyme dosages on physical properties of pulps.

Pulps	Enzyme dosage (IU/g)	Beating degree (°SR)	Tensile index (N·m/g)	Burst index (kPa·m^2^/g)	Tear index (mN·m^2^/g)
Aspen CMP	Control	19.0 ± 0.04	15.48 ± 0.02	0.71 ± 0.04	1.54 ± 0.03
	0.20	19.0 ± 0.02	15.46 ± 0.01	0.71 ± 0.03	1.55 ± 0.02
	0.60	18.0 ± 0.05	17.20 ± 0.01	0.85 ± 0.04	1.77 ± 0.04
	0.80	17.5 ± 0.01	14.65 ± 0.01	0.68 ± 0.05	1.48 ± 0.01
Pine CMP	Control	11.0 ± 0.04	20.90 ± 0.06	1.46 ± 0.07	3.03 ± 0.08
	0.20	11.0 ± 0.04	20.90 ± 0.04	1.46 ± 0.06	3.02 ± 0.07
	0.60	10.5 ± 0.07	21.77 ± 0.07	1.51 ± 0.08	3.19 ± 0.03
	0.80	10.0 ± 0.05	19.43 ± 0.03	1.46 ± 0.02	3.00 ± 0.02
Wheat straw CP	control	20.0 ± 0.01	26.71 ± 0.05	1.74 ± 0.01	3.64 ± 0.04
	0.20	17.0 ± 0.03	30.83 ± 0.06	2.03 ± 0.01	4.07 ± 0.02
	0.60	16.5 ± 0.04	28.45 ± 0.02	1.89 ± 0.02	3.88 ± 0.01
	0.80	16.0 ± 0.04	24.23 ± 0.01	1.69 ± 0.08	3.42 ± 0.02

*Enzyme treatment conditions: pH 7.0, temperature 55 °C, 10% pulp consistency and treatment time 2 h.

Compared to hardwood pulp and softwood pulp, however, beating degree of wheat straw pulp was effectively decreased by treatment with the recombinant EG at a low enzyme dosage (0.2 IU/g). Meanwhile, the tensile index, burst index and tear index increased to 30.83 N·m/g, 2.03 kPa·m^2^/g and 4.07 mN·m^2^/g respectively, which were 15.4%, 16.9% and 11.8% higher than the control (without enzymatic treatment) correspondingly. Similarly, more enzyme dosage could result in poor strength properties of wheat straw pulp due to the fiber damage.

Based on above, it was verified that the alkali-tolerant EG produced by engineering strain Y106OE-EG could be used for modification of both wood pulp and non-wood pulp, which resulted in lower beating degree and higher pulp strength. Relatively, the recombinant EG was most effective for improving properties of wheat straw chemical pulp.

#### Effect of pH and temperature on pulp modification

Different pH (pH 6.0 to pH 10.0) and temperature (45 °C to 75 °C) were used in enzymatic modification to evaluate their effect on enzymatic modification, and the results were showed in Tables 2 and 3. As shown in Table 2, Enzymatic treatment with the recombinant EG could improve pulp drainage and strength properties within a wide range of pH 6.0 to 10.0, but more effective in the range of pH 6.0 to 8.0. The most efficient treatment was obtained at pH 7.0 for all types of pulps, which was similar to the optimal pH of the recombinant EG (Fig. 2a). Enzymatic treatment at higher than pH 9.0 led to the pulps strength index decrease.

**Table 2:** Effect of different pH during enzyme treatment on pulp properties.

Pulps	pH	Beating degree (°SR)	Tensile index (N·m/g)	Burst index (kPa·m^2^/g)	Tear index (mN·m^2^/g)
Aspen CMP	Control	19.0 ± 0.04	15.48 ± 0.02	0.71 ± 0.04	1.54 ± 0.03
	6.0	18.0 ± 0.07	17.25 ± 0.04	0.85 ± 0.06	1.77 ± 0.02
	7.0	18.0 ± 0.05	17.22 ± 0.01	0.85 ± 0.05	1.76 ± 0.04
	8.0	18.0 ± 0.04	17.18 ± 0.01	0.82 ± 0.02	1.75 ± 0.02
	9.0	18.5 ± 0.09	16.70 ± 0.06	0.76 ± 0.01	1.67 ± 0.05
	10.0	19.0 ± 0.02	15.94 ± 0.02	0.72 ± 0.01	1.58 ± 0.01
Pine CMP	Control	11.0 ± 0.04	20.90 ± 0.06	1.46 ± 0.07	3.03 ± 0.08
	6.0	10.5 ± 0.03	21.74 ± 0.04	1.51 ± 0.03	3.19 ± 0.04
	7.0	10.5 ± 0.07	21.79 ± 0.07	1.50 ± 0.08	3.18 ± 0.03
	8.0	10.5 ± 0.02	21.70 ± 0.08	1.50 ± 0.04	3.19 ± 0.05
	9.0	11.0 ± 0.06	21.04 ± 0.06	1.48 ± 0.01	3.09 ± 0.02
	10.0	11.0 ± 0.04	20.90 ± 0.02	1.47 ± 0.02	3.05 ± 0.06
Wheat straw CP	Control	20.0 ± 0.01	26.71 ± 0.05	1.74 ± 0.01	3.64 ± 0.04
	6.0	17.0 ± 0.02	30.85 ± 0.03	2.02 ± 0.03	4.05 ± 0.01
	7.0	17.0 ± 0.04	30.83 ± 0.06	2.03 ± 0.01	4.07 ± 0.02
	8.0	17.0 ± 0.04	29.96 ± 0.02	2.00 ± 0.07	3.83 ± 0.05
	9.0	17.5 ± 0.01	28.47 ± 0.02	1.84 ± 0.09	3.77 ± 0.02
	10.0	19.0 ± 0.06	26.94 ± 0.06	1.79 ± 0.02	3.65 ± 0.02

*Enzyme dosage: 0.2 IU/g for wheat straw pulp, and 0.6 IU/g for hardwood pulp and softwood pulp, based on CMCase activity and oven dry pulp weight. **Other conditions of enzyme treatment: 55 °C, 10% pulp consistency and treatment time 2 h.

**Table 3:** Effect of different temperature during enzymatic treatment on pulp properties.

Pulps	Temperature (°C)	Beating degree (°SR)	Tensile index (N·m/g)	Burst index (kPa·m^2^/g)	Tear index (mN·m^2^/g)
Aspen CMP	CK	19.0 ± 0.04	15.48 ± 0.02	0.71 ± 0.04	1.54 ± 0.03
	45	18.0 ± 0.03	17.20 ± 0.03	0.80 ± 0.03	1.70 ± 0.04
	55	18.0 ± 0.05	17.72 ± 0.01	0.85 ± 0.05	1.76 ± 0.04
	65	18.5 ± 0.07	16.94 ± 0.01	0.79 ± 0.08	1.68 ± 0.02
	75	19.0 ± 0.04	16.02 ± 0.03	0.75 ± 0.04	1.60 ± 0.05
Pine CMP	CK	11.0 ± 0.04	20.90 ± 0.06	1.46 ± 0.07	3.03 ± 0.08
	45	11.0 ± 0.03	21.72 ± 0.02	1.49 ± 0.01	3.11 ± 0.02
	55	10.5 ± 0.07	22.29 ± 0.07	1.50 ± 0.08	3.17 ± 0.04
	65	11.0 ± 0.09	21.35 ± 0.05	1.48 ± 0.03	3.16 ± 0.01
	75	11.0 ± 0.01	21.87 ± 0.02	1.48 ± 0.02	3.08 ± 0.06
Wheat straw CP	CK	20.0 ± 0.01	26.71 ± 0.05	1.74 ± 0.01	3.64 ± 0.04
	45	17.5 ± 0.02	28.98 ± 0.03	1.93 ± 0.02	3.88 ± 0.05
	55	17.0 ± 0.04	30.83 ± 0.06	2.03 ± 0.01	4.07 ± 0.02
	65	17.0 ± 0.07	29.77 ± 0.09	1.90 ± 0.01	3.94 ± 0.01
	75	18.5 ± 0.05	27.40 ± 0.01	1.79 ± 0.05	3.72 ± 0.07

*The pulps were treated at pH 7.0, and other conditions were same as Table 2.

Table 3 showed that, compared to the control, the recombinant EG treatment in the range of 45 °C to 65 °C slightly reduced the beating degree of aspen CMP and well improved the aspen CMP strength properties (tensile index, burst index and tear index), but almost not affected the beating degree of pine CMP when compared with the control, except treatment with EG at 55 °C slightly decreased beating degree of pine CMP. The properties of wheat straw CP could be effectively improved after the recombinant EG treatment in the range of 45 °C to 65 °C, and the temperature of 55 °C was optimum, which was identical with the optimum temperature of the recombinant EG (Fig. 2b).

### Possible mechanism of pulp modification with recombinant EG from engineering strain *Bacillus subtilis* Y106OE-EG

#### Changes in fiber characteristics of different pulps after enzymatic treatment

Table 4 showed that the mean fiber length of aspen CMP was increased after the recombinant EG treatment, but the mean width was almost no change when compared with the control. Thus the ratio of fiber length to fiber width was increased, which was beneficial for improving breaking length and tear strength of the pulp. However, there were only little differences in fiber characteristics between the EG-treated pine pulp and the control, possibly due to that: (1) high hemicellulose content in the softwood pulp affected the accessibility of enzyme^33, 34^; (2) compared to the hardwood and non-wood pulp, the softwood pulp fiber cells have more compact structure, which hindered the enzyme access to fiber.

**Table 4:** Physical characteristics of fiber in various pulps treated with the EG from engineering strain Y106OE-EG.

Pulps type		Fines content (%)	Fiber length (mm)	Fiber width (μm)	Curl index	Kink index
Aspen CMP	Control	3.67 ± 0.03	1.74 ± 0.02	18.72 ± 0.05	0.13 ± 0.07	1.67 ± 0.03
	Treated with EG	3.60 ± 0.02	1.80 ± 0.08	18.70 ± 0.03	0.11 ± 0.03	1.59 ± 0.05
Pine CMP	Control	4.50 ± 0.04	2.25 ± 0.02	26.39 ± 0.02	0.17 ± 0.02	0.46 ± 0.03
	Treated with EG	4.48 ± 0.05	2.26 ± 0.01	26.40 ± 0.03	0.17 ± 0.03	0.43 ± 0.07
Wheat straw CP	Control	10.30 ± 0.03	0.89 ± 0.03	19.20 ± 0.03	0.20 ± 0.06	1.09 ± 0.02
	Treated with EG	9.68 ± 0.01	1.02 ± 0.04	18.98 ± 0.01	0.14 ± 0.08	0.88 ± 0.02

The presence of fines affects paper strength by influencing bonding of fibers. The curly fibres do also distribute the damages over a larger area and made more bonds broken, which might ultimately lead to lower paper strength. It could also be found that, after the EG treatment, the amount of fines in wood pulps, the curl index and kink index were decreased compared to the control respectively, which were beneficial for improving paper strength. Relatively, the changes of fines amount, curl index and kink index of hardwood pulp were more than that of softwood pulp, which should be part reasons that the EG treatment was more effective for hardwood pulp than for softwood pulp.

Wheat straw pulp had lower mean length and width of fiber, and higher content of fines compared to wood pulps. After the EG treatment, the mean length of fibers in wheat straw pulp increased but the mean width decreased, suggesting that the ratio of length to width increased obviously. The curl index and kink index of wheat straw pulp were also decreased after enzymatic treatment with recombinant EG, which would conducive to the paper strength improvement. High fine contents in straw pulp were believed to damage the drainage property and decrease the paper strength by affecting combination of fibers, which was also the main obstacle for straw pulp application in papermaking industry. Table 4 showed that, the EG treatment reduced the content of tiny fiber that existing in wheat straw pulp, and relatively increased the ratio of long fibers, thus resulted in improvement of drainage and strength properties of wheat straw pulp. This should also be a reason why enzymatic treatment was more effective for wheat straw pulp modification than the wood pulps.

#### Change in HBI and crystallinity

Table 5 showed that, after treatment with the recombinant EG, the HBI values of all the pulps were increased when compared with the control respectively, indicated that EG treatment increased hydrogen bonding of the fibers in these pulps, which help to improve the pulp strength properties. Relatively, the HBI of pine CMP was just slightly increased, which was in accordance with the slight influence on the pulp strength.

**Table 5:** HBI and crystallinity of different pulps before and after enzymatic treatment.

Pulps		Crystallinity (%)	HBI
Aspen CMP	Control	34.5	1.88
	Treated with EG	36.8	2.06
Pine CMP	Control	46.1	1.60
	Treated with EG	46.3	1.71
Wheat straw CP	Control	37.4	1.04
	Treated with EG	41.3	1.37

The crystallinity of cellulose is a critical parameter that demonstrated supramolecular structure of cellulose. The hydroxyl groups in the crystalline region could form intramolecular hydrogen bond and intermolecular hydrogen bond, which contributed to the fiber strength. As shown in Table 5, the degree of crystallinity of cellulose increased from 34.5% to 36.8% for aspen CMP, and from 37.4% to 41.3% for wheat straw CP after enzymatic treatment with the recombinant EG when compared to the control, but almost no change for pine pulp. The results indicated that the EG treatment was more helpful to improve the strength properties of hardwood pulp and non-wood pulp compared with softwood pulp.

## Materials and Methods

### Plasmids, strains and materials

*Bacillus subtilis* Y106-WT was isolated from alkaline soil and maintained in our laboratory^21^. The *E*.*coli*/*B*.*subtilis* shuttle vector pHT43 was obtained from Invitrogen (America) and used for protein expression.

Three types of pulps were used in this study. In which, aspen CMP was provided by Huatai Group Corp. Ltd (China), and chemical compositions of the pulp were cellulose of 35.0%, hemicellulose of 9.5%, and lignin of 25.5%; the pine CMP with the chemical compositions of 40.0% cellulose, 14.5% hemicellulose and 18.5% lignin was stored in our lab; wheat straw chemical pulp was from Qranlin Group Co.(China), and its chemical composition was cellulose of 61.0%, hemicellulose of 15.6%, and lignin of 4.8%. All the pulps were firstly washed by tap water to remove water soluble impurity, and store at 4 °C for further use.

### Construction and transformation of the plasmid pHT43-Y106-EG for EG expression

The *E*. *coli*/*B*. *subtilis* shuttle vector pHT43 was used for cloning and expressing the alkali-tolerance EG (Y106-EG). It was constructed based on the map of vector which was showed in Fig. 1a. Standard techniques were used for nucleic acid manipulations. Briefly, the Y106-*eg*(GenBank: AF355629.1) gene (without signal peptide, with native Y106-EG terminator and His-Tag) was cloned from the genome of *Bacillus subtilis* Y106-WT. The gene-specific primers using in polymerase chain reaction (PCR) were showed as follows: *eg*5′TACAAAAACATCAGCCGTAGGATCCGCAGGGACAAAAACGCCAGTAGC3′(forwardprimer) and 5′ CTAATGGTGATGGTGATGATGATTTGGTTCTGTTCCCCAAA 3′ (reverse primer).

*eg*-T5′ATCATCACCATCACCATTAGTTAAGCTTTTTTTGGCGGAC3′forwardprimer) and 5′ CCCGGGGACGTCGACTCTAGAACGGCTTCTAAATCTTCCAG3′ (reverse primer).

The PCR amplification was executed with following steps: the first step was at 94 °C for 2 min, followed by 30 cycles of 98 °C for 10 s, 58 °C for 30 s, and 68 °C for 2 min, and the final extension was carried out at 72 °C for 5 min. The products were separated by electrophoresis. Then, 1.71 kb of fragment for EG gene was purified from gel using the TIANgelMidi Purification Kit (Tiangen Biotech, Beijing, China).

The expression element of plasmid pHT43 (Fig. 1a) were an inducible promoters P*groE* ^22^ and a signal peptide S*amy*Q which conducted the expression of Y106-*eg* gene. The plasmid pHT43 was firstly digested for 2 h at 37 °C with FastDigest^®^ B*am*HI (Fermentas, Canada) and FastDigest^®^ X*ba*I (Fermentas, Canada). Then, fragment of 1.71 kb was sub-cloned into the digested vector by recombinant enzyme (pEASY^®^-Uni Seamless Cloning and Assembly Kit, Trans Gen Biotech, China). The recombinant plasmid pHT43-Y106-EG was identified by restriction analysis and sequencing.

The recombinant plasmid pHT43-Y106-EG was transformed into the competent cells prepared from *B. subtilis* Y106-WT, according to the previous transformation protocol reported by Xue *et al*.^35^. The transformants were selected by culturing 12 h at 37 °C on the LB agar plates containing 5 μg/ml chloromycetin. To ensure the enzyme activities, the colonies were cultured in 50 mL of fermentation medium at 37 °C for 72 h in shake flask (300 mL).

### Purification and identification of recombinant EG

The fermentation broths of Y106OE-EG was concentrated, and loaded onto an HisTrapTMFF crude column (GE Healthcare, Sweden), then, eluted with elution buffer containing 300 mM imidazole, 500 mM NaCl and 20 mM NaH_2_PO_4_-K_2_HPO_4_ (pH 7.0) to obtain pure EG by His-Tag.

Sodium dodecyl sulfate polyacrylamide gel electrophoresis (SDS-PAGE) was used to analyze the subunit molecular weight of recombinant EG, and finally the recombinant protein was identified by mass spectrometry.

### Effect of pH and temperature on the activity and stability of recombinant EG

Optimal pH of purified recombinant EG from Y106OE-EG was determined by measuring its enzyme activity with 2% (w/v) carboxymethyl-cellulose(CMC) in 55 °C for 30 min, at pH range of 4.0 to 10.0.

pH stability for purified EG was determined by incubating the enzyme in buffer of different pH values (pH 4.0 to 9.0) for 12 h at 4 °C, then measuring the CMCase activity with 2% (w/v) CMC in pH 6.5 at 55 °C.

The buffers used to establish the optimum pH and to assess pH stability were as follows: sodium acetate buffer(0.2 mol/L NaAc and 0.2 mol/L HAc, pH 4.0–5.0), phosphate buffer (0.06 mol/L Na_2_HPO_4_ and 0.06 mol/L KH_2_PO_4_, pH 6.0–8.0) and Gly-NaOH buffer (0.2 mol/L glycine and 0.2 mol/L NaOH, pH 9.0–10.0).

Optimal temperature was determined by measuring the CMCase activity with 2% (w/v) CMC in buffer solution (pH 6.5) at different temperature of 40 °C to 70 °C.

The temperature stability was determined by measuring the CMCase activity with 2% (w/v) CMC in pH 6.5 buffer after incubation from 40 °C to 70 °C for 60 min.

### Production of EG by engineering strain *Bacillus subtilis* Y106OE-EG in shake flask

The engineering strain Y106OE-EG was firstly cultured in bran medium at 37 °C, 200 rpm for 6 h (added 10ug/ml chloromycetin), then cultured with IPTG addition (0.1 mM) for 66 h at 28 °C in 300 ml shake flask containing 50 ml of bran medium.

Bran medium (bran 50 g/L, peptone 2.5 g/L, NaCl 5 g/L, K_2_HPO_4_ 1 g/L, MgSO_4_·7H_2_O 0.2 g/L, pH 7.0) was used as the culture medium for liquor fermentation, which was optimized by Chen *et al*. previously^18^.

The seed medium used for culturing *E*. *coli* and *B*. *subtilis* was the Luria-Bertani media (LB media, 10 g/L peptone, 5 g/l yeast extract, and 5 g/l NaCl, pH 7.0).

### Enzymatic modification of pulps

Enzymatic modification of pulp were executed with 15 g (dry weight) of pulp at 10% (w/v) pulp consistency in phosphate buffer solution(0.06 mol/LNa_2_HPO_4_ and 0.06 mol/LKH_2_PO_4_, pH 6.0–8.0) and Gly-NaOH buffer (0.2 mol/L glycine and 0.2 mol/L NaOH, pH 9.0–10.0). Enzymes were firstly added to the buffer, and then to the pulp for a well distribution. Enzymatic treatment was carried out in plastic bag in a water bath at 55 °C for 2 h. The sample was taken out periodically and kneaded for 10~15 seconds for homogeneous reaction. After enzymatic treatment, the pulp was placed in water bath at 90 °C for 1 h to terminate the reaction, then filtered and washed with deionized water. The control was performed by the same process but no adding enzyme.

### Preparation of handsheets

Handsheets (basis weight of 60 ± 2 g/m^2^) were prepared using a Blattbildner-Sheet former (RK-3A, Austria)^36, 37^, then, stored at the temperature humidity chamber overnight for strength properties testing.

### Analytical methods

#### Assays of enzyme activities and biomass

The CMCase activity for 30 min was measured with 2% w/v sodium carboxymethyl-cellulose (Sigma, USA), according to the method described previously^38^. The filter paper activity (FPA) was measured with Whatman^TM^ 1# filter papers(0.5 g of substrate), incubated at 50 °C for 1 h, according to Ghose *et al*.^39^.

The unit of CMCase activity and FPA was defined as the amount of enzyme that released 1 μmol of reducing sugar per min under the relevant assay conditions.

Biomass analysis was based on the nuclear quantity of the culture described previously^40^.

#### Analysis of pulp strength properties

The tensile index, burst index, and tear index of the handsheets were analyzed with a tensile strength tester (ZL-100A, China), bursting strength tester (BSM-1600B, China), and tearing tester (SLY-1000, China) according to the Chinese standard methods (GB/T 12914-2008, GB/T 454-2002 and GB/T 455-2002) respectively, which described previously^41^.

#### Analysis of beating degree of pulp

Beating degree of pulp was tested using beating degree tester (PTI Austria) according to the Schopper-Riegler method (GB/T3332-1982). The beating degree value was calculated according to the following formula (1) described previously^41^

1 $${\rm{Beating}}\,{\rm{degree}}=(\mathrm{1000}-{\rm{V}})/\mathrm{10}\,(^\circ \mathrm{SR})$$

in which, V: volume of filtrate discharge from sideway-duct.

#### Analysis of fiber characteristics

The fiber characteristics such as fines content, fiber length, fiber width, curl index and kink index were measured with fiber quality analyzer (FQA, Optest Equipment Inc, Canada). The fines were defined as the fiber portion with fiber length between 70 μm to 200 μm.

#### Analysis of hydrogen-bond intensity and crystallinity

FTIR-ATR spectra of pulps were recorded with a Tensor 27 spectrophotometer (Brüker, Germany) under the following conditions: ZnSe crystals, 45° angle of incidence, 2 cm resolution, 32 independent scans, and scan range of 4000 cm^−1^ to 650 cm^−1^.

The relative absorbance ratio (A4000~2995/A1337) represented the decrease of absorbance as a criterion of hydrogen-bond intensity (HBI)^42, 43^.

Crystallinity of cellulose was tested using X-ray diffraction (XRD). XRD analysis was executed with a D8 Powder X-ray Diffractometer (Bruker AXS, Germany), which was equipped with a CuXa X-ray tube. The experimental conditions were used as follows: 2*θ* = 5°~50° and step-scan of 2*θ* = 0.5°, and calculated according to the formula (2) as follows^44–46^

2 $${\rm{CRI}}=\frac{{{\rm{I}}}_{002}-{{\rm{I}}}_{{\rm{am}}}}{{{\rm{I}}}_{002}}\times 100$$

in which, CRI is the crystallinity index; I_002_ is the intensity of the crystalline peak at the maximum at 2*θ* between 22° and 23° for cellulose, and I_am_ is the intensity at the minimum at 2*θ* between 18° and 19° for cellulose.

### Statistical analysis

A *t*-Student one-tail test was carried out with the software Microsoft Office 2013 Excel (Microsoft, USA). The mean data, standard deviations, and *P* values were calculated in all quantitative analysis.

## Conclusions

An alkali-tolerant EG gene over-expressed strain *Bacillus subtilis* Y106OE-EG was successfully constructed and more cellulase with high EG activity of 8.45 IU/ml but little FPA of 0.12 IU/ml was produced by liquor fermentation. The optimum pH and temperature for the recombinant EG was pH 6.5 and 55 °C respectively, and the EG was stable at pH value of 4.0 to 9.0 and temperature of 40 °C to 60 °C, which indicated it was more suitable for alkaline environment in pulping industry than acidic cellulases often used in industrial process now. Treatment with the EG could reduce the beating degree, enhance drainage and strength properties of wood pulp and non-wood pulp, especially for wheat straw chemical pulp, which indicated a good potential application in pulp modification. The increase in ratio of fiber length to width and cellulose crystallinity, enhancement of fibers binding by hydrogen bond, and decrease in fine contents and curl index were considered to be some reasons for improvement of pulp properties after the EG treatment.
